# Populations of inhibitory and excitatory interneurons in lamina II of the adult rat spinal dorsal horn revealed by a combined electrophysiological and anatomical approach

**DOI:** 10.1016/j.pain.2010.08.008

**Published:** 2010-11

**Authors:** Toshiharu Yasaka, Sheena Y.X. Tiong, David I. Hughes, John S. Riddell, Andrew J. Todd

**Affiliations:** Neuroscience and Molecular Pharmacology, Faculty of Biomedical and Life Sciences, University of Glasgow, Glasgow G12 8QQ, UK

**Keywords:** Whole cell patch-clamp recording, GABA, Glutamate, Firing pattern, A-current, Somatostatin

## Abstract

Lamina II contains a large number of interneurons involved in modulation and transmission of somatosensory (including nociceptive) information. However, its neuronal circuitry is poorly understood due to the difficulty of identifying functional populations of interneurons. This information is important for understanding nociceptive processing and for identifying changes that underlie chronic pain. In this study, we compared morphology, neurotransmitter content, electrophysiological and pharmacological properties for 61 lamina II neurons recorded in slices from adult rat spinal cord. Morphology was related to transmitter content, since islet cells were GABAergic, while radial and most vertical cells were glutamatergic. However, there was considerable diversity among the remaining cells, some of which could not be classified morphologically. Transmitter phenotype was related to firing pattern, since most (18/22) excitatory cells, but few (2/23) inhibitory cells had delayed, gap or reluctant patterns, which are associated with A-type potassium (*I*_A_) currents. Somatostatin was identified in axons of 14/24 excitatory neurons. These had variable morphology, but most of those tested showed delayed-firing. Excitatory interneurons are therefore likely to contribute to pain states associated with synaptic plasticity involving *I*_A_ currents. Although noradrenaline and serotonin evoked outward currents in both inhibitory and excitatory cells, somatostatin produced these currents only in inhibitory neurons, suggesting that its pro-nociceptive effects are mediated by disinhibition. Our results demonstrate that certain distinctive populations of inhibitory and excitatory interneuron can be recognised in lamina II. Combining this approach with identification of other neurochemical markers should allow further clarification of neuronal circuitry in the superficial dorsal horn.

## 1 Introduction

Lamina II of the dorsal horn [46] is a major target for nociceptive primary afferents [26,54] and has long been known to play a role in modulating incoming sensory information [35]. It contains a high density of small neurons, and virtually all of those in lumbar segments have axons that remain within the spinal cord [65]. Immunocytochemical studies have shown that approximately 30% of lamina II neurons are GABA-immunoreactive and that a subset of these are enriched with glycine [40,61]. The remaining neurons appear to be glutamatergic, based on their expression of vesicular glutamate transporter 2 (VGLUT2) [32,55]. The region contains a variety of neuromodulators, including neuropeptides (expressed by local neurons and primary afferents) and monoamines that are released by axons descending from the brainstem [56].

The neuronal organisation of the superficial dorsal horn is very complex and an important pre-requisite for our understanding of its circuitry is a comprehensive classification scheme for lamina II interneurons. Several studies have attempted to classify these cells based on their morphology, revealed with the Golgi technique [3,9,10,57], trans-synaptic labelling [6], intracellular injection *in vivo*[4,27,45,66] or labelling obtained in *in vitro* preparations [1,14,16,17,28–30,32,33,62,67,69]. In addition, cells have been characterised according to their firing patterns in response to current injection [7,12,14,17,18,50,62,68], and based on their expression of neuropeptides, receptors and other neurochemical markers [56,59].

The most widely accepted scheme has emerged from the pioneering studies of Perl and co-workers [14], who identified 4 main types of neuron: islet and central cells, both with rostrocaudally elongated dendrites, and radial and vertical cells with dendrites that have significant dorsoventral extension. However, there are important unresolved issues concerning the classification of lamina II neurons. First, the relationship between morphology and transmitter content is likely to be complex, since both GABAergic and glutamatergic cells have been found among central, radial and vertical populations [16,17,29,32]. Second, it has been difficult to find correlations between firing pattern and either morphology [7,12,17,34,62], or neurotransmitter type [17,50]. Third, there is limited information about the responses of inhibitory or excitatory interneurons to different neuromodulators.

The purpose of this study was to identify functional populations by investigating a sufficiently large sample of well-characterised lamina II neurons in adult rat spinal cord. Our strategy was to compare electrophysiological properties (in particular, firing pattern in response to current injection) with morphology and to use neurotransmitter content to identify excitatory and inhibitory interneurons. In order to investigate whether responses to neuromodulators are associated with particular types of neuron we also examined responses to noradrenaline (NA) and 5HT, which have been shown to act on the majority of lamina II neurons [1,15,30,38], and to somatostatin, which causes outward currents in many of these cells [21,23]. The latter was of particular interest, as there is immunocytochemical evidence that the somatostatin 2a (sst2a) receptor is only expressed by GABAergic neurons in this region [60]. Since somatostatin is synthesised by certain excitatory interneurons in the superficial dorsal horn [43] we also tested the axons of some of these cells for the peptide.

## 2 Methods

### 2.1 Ethical approval

All experiments adhered to the guidelines of the Committee for Research and Ethical Issues of IASP. They were approved by the Ethical Review Process Applications Panel of the University of Glasgow and were performed in accordance with the UK Animals (Scientific Procedures) Act 1986.

### 2.2 Animals and slice preparation

The methods used for obtaining spinal cord slices from adult rats were similar to those previously described [67]. Briefly, 41 male Wistar rats (6–10 weeks old) were deeply anaesthetized with halothane or isoflurane. After thoracolumbar laminectomy, the spinal cord was removed into ice-cold dissection solution (mM: NaCl 0, KCl 1.8, KH_2_PO_4_ 1.2, CaCl_2_ 0.5, MgCl_2_ 7, NaHCO_3_ 26, glucose 15, sucrose 254, oxygenated with 95% O_2_, 5% CO_2_). The rats were then killed by anaesthetic overdose and decapitation. In most cases, all dorsal and ventral roots were removed but in some cases one or more dorsal roots were kept intact. Spinal cords were then embedded in low-melting-point agarose and cut into 500 μm thick parasagittal or horizontal slices with a VT1000S vibrating blade microtome (Leica Microsystems Ltd., Milton Keynes, UK). The slices were stored in dissection solution or transferred directly to a recovery chamber where they were perfused with normal Krebs’ solution (identical to the dissection solution except for (mM): NaCl 127, CaCl_2_ 2.4, MgCl_2_ 1.3 and sucrose 0) at room temperature. Parasagittal and horizontal slices were used in this study since these are more likely to preserve the dendritic trees of lamina II neurons, which are often elongated in the rostrocaudal axis.

### 2.3 Patch-clamp recording

A single slice was transferred to a recording chamber and perfused with normal Krebs’ solution at 10 ml min^−1^ at room temperature. Slices that had been stored in dissection solution were perfused for at least 30 min before recording. Lamina II was identified as a translucent band across the dorsal horn under a dissecting microscope. Blind whole-cell voltage- or current-clamp recordings were made from neurons in this region as previously described [67], by using glass pipettes (7–12 MΩ) filled with a solution containing the following (mM): potassium gluconate 120, KCl 20, MgCl_2_ 2, Na_2_ATP 2, NaGTP 0.5, Hepes 20, EGTA 0.5, and 0.2% Neurobiotin (pH 7.28 adjusted with KOH). Signals were acquired with a patch-clamp amplifier (Axopatch 200B or MultiClamp 700B, Molecular Devices, Sunnyvale, CA) and acquisition software (pCLAMP 9 or 10, Molecular Devices). Signals were low-pass filtered at 5 kHz, amplified 10-fold in voltage-clamp mode or 50-fold in current-clamp mode, sampled at 10 kHz and analysed offline using pCLAMP 9 or 10. No correction for the liquid junction potential was made.

### 2.4 Passive membrane properties

The resting membrane potential was determined immediately after establishing the whole-cell configuration. Neurons that had a resting membrane potential less negative than −40 mV were not used for electrophysiological recording. We set this relatively low value because the mean resting potential for islet cells reported by Grudt and Perl [14] was −47.7 mV. However, we found that all but one of the cells in our sample had a resting membrane potential more negative than −50 mV. The built-in pCLAMP membrane test was used to monitor membrane properties during recording.

### 2.5 Protocol for assessing discharge pattern and active membrane properties

The protocol used to test firing patterns in this study was based on that described by Sandkühler and co-workers [17,47,48]. Because firing pattern appears to depend on holding potential [17,47], we used a standardised protocol that involved testing each cell from three different potentials (one from between −50 and −65 mV, one from between −65 and −80 mV and one from a potential more negative than −80 mV). Firing patterns were determined in response to depolarising current injections of 1 s duration from each of these potentials, in order to determine any voltage dependence.

A voltage step protocol was used to assess the presence of A-type potassium currents (A-currents, *I*_A_), hyperpolarisation-activated currents (H-currents, *I*_h_), and currents through low threshold calcium channels (*I*_Ca_). The membrane potential was held at −50 mV (or in some cases at −40 mV) and increasing negative voltage steps of 1 s duration were applied (usually over the range −60 to −140 mV, with 10 mV steps).

### 2.6 Drug application

The actions of NA (bitartrate salt, 20–40 μM; Sigma–Aldrich, Gillingham, UK), 5HT (hydrochloride salt, 10–40 μM; Sigma–Aldrich) and somatostatin (1–2 μM, Merck Chemicals Ltd., Nottingham, UK) were tested by bath application for 1 min on some of the neurons. The drugs were dissolved in Krebs solution and were applied via three-way stopcocks without any change in either perfusion rate or temperature. The choice of doses for each drug was based on those found to be effective in previous studies [21,23,30].

### 2.7 Tissue processing, immunocytochemistry and confocal microscopy

Following completion of whole-cell patch-clamp recording, the slices were fixed overnight in 4% formaldehyde in 0.1 M phosphate buffer at 4 °C. Slices were rinsed in phosphate buffer, flat embedded in 3% agar and were then cut into parasagittal 60 μm-thick sections with a Vibratome. Free-floating sections were immersed in 50% ethanol for 30 min to enhance antibody penetration and then incubated overnight in avidin–rhodamine (1:1000; Jackson ImmunoResearch, West Grove, PA) to reveal the Neurobiotin-labelled neurons.

All sections that contained labelled profiles were scanned with a MRC1024 confocal microscope (Bio-Rad, Hemel Hempstead, UK) with a Krypton–Argon laser. Overlapping fields from each section were scanned through a 40 × oil-immersion lens with a *z*-separation of 1 μm.

To determine the neurochemical phenotype of each of the labelled cells, selected sections that contained axonal boutons from the cell were rinsed several times in PBS and were then re-incubated for 3 days in a cocktail of guinea pig antibody raised against VGLUT2 (1:5000; Millipore, Watford, UK) and rabbit antibody raised against vesicular GABA transporter (VGAT; 1:1000; Synaptic Systems, Goettingen, Germany). This was followed by incubation in species-specific donkey secondary antibodies: anti-guinea pig IgG conjugated to cyanine 5.18 (Cy5; 1:100; Jackson ImmunoResearch) and anti-rabbit IgG conjugated to Alexa 488 (1:500; Invitrogen, Paisley, UK). All antibodies and avidin–rhodamine were diluted in PBS that contained 0.3 M NaCl and 0.3% Triton X-100. On completion of immunocytochemical reactions, all sections were mounted in anti-fade medium (Vectashield; Vector Laboratories, Peterborough, UK) and stored at −20 °C. Antibody against VGAT was used to identify inhibitory neurons, rather than antibodies against either of the isoforms of the GABA-synthesising enzyme glutamate decarboxylase (GAD), because we have found that levels of GAD65 or GAD67 in some GABAergic boutons in the dorsal horn are very low and this can make it difficult to confirm their identity [31]. Although VGAT is present in both GABAergic and glycinergic boutons [5], we have provided evidence that virtually all glycinergic neurons in lamina II are also GABAergic [40,61].

For each of the recorded cells, one or two sections that contained part of the axon and had been immunoreacted (as described above) were scanned with a Radiance 2100 confocal microscope (Bio-Rad) equipped with Argon, 543 nm green HeNe and 638 nm red diode lasers, through a 60 × oil-immersion lens with a *z*-separation of 0.3 or 0.5 μm. In order for a cell to be classified as excitatory or inhibitory, at least 6 of its axonal boutons had to display immunoreactivity for VGLUT2 or VGAT, respectively. None of the cells with VGLUT2-positive boutons showed staining for VGAT, and *vice versa*.

For 24 of the cells that had VGLUT2-immunoreactive axons, additional sections that contained parts of the axonal arborisation were available, and these were incubated for 3 days in rabbit antiserum against somatostatin (Bachem, St. Helens, UK; 1:1000) and then overnight in donkey anti-rabbit IgG conjugated to Alexa 488. Sections were scanned with the Radiance confocal microscope (as described above) to look for somatostatin immunoreactivity in Neurobiotin-labelled axonal boutons. Again, at least 6 boutons had to display immunoreactivity for the cell to be defined as somatostatin positive.

The VGAT antibody (catalogue number 131002, lot #20) was raised against a peptide corresponding to amino acids 75–87 of the rat VGAT, and staining was abolished by pre-incubation of the antibody with the immunising peptide at 10^−6^ M (data not shown). The guinea pig antibodies against VGLUT2 (catalogue number AB5907 lot #23100234 and catalogue number AB2251 lot #NG1554203) were raised against a peptide from the rat VGLUT2. The specificity of both antibodies was confirmed by carrying out dual immunofluorescence staining with a well-characterised rabbit antibody against VGLUT2 [55]. In each case, identical structures were stained by the rabbit and guinea pig antibodies (data not shown). The somatostatin antibody (catalogue number T-4103; lot #A03606) was raised against somatostatin-14, and shows 100% cross reaction with longer forms of the peptide (somatostatin −25 or −28), but none with substance P, neuropeptide Y (NPY) or vasoactive intestinal polypeptide (manufacturer’s specification).

### 2.8 Morphological reconstruction and analysis

Projections of confocal image stacks that included the cell body, dendrites and axon of each neuron were used to reveal morphology. These were viewed in Adobe Photoshop 10 (Adobe Systems, San Jose, CA) and all labelled profiles were selected and pasted onto a black background. Corresponding regions from different Vibratome sections were orientated by aligning cut surfaces of dendrites and axons, and images from these different sections were then superimposed. The laminar locations of the cell bodies were determined by using dark field microscopy. As described previously [14,60,67], the superficial dorsal horn (laminae I and II) appears as a distinctive dark band under these conditions, due to the relative lack of myelinated fibres. However, the thickness of lamina I varies considerably, being thickest in the central part [60], while the border between laminae I and II is often difficult to determine. In this study, we therefore defined lamina II as a 100 μm thick band extending dorsally from the II/III border, and this was divided into three equal parts. The superficial and deep thirds were defined as IIo and IIi, respectively, while the middle third was defined as the border between IIo and IIi, in order to allow direct comparison with the results of Grudt and Perl [14] and Yasaka et al. [67]. Dimensions of dendritic arbors in the rostrocaudal and dorsoventral planes were measured from the superimposed image stacks.

### 2.9 Statistical tests

The unpaired *t*-test or Chi-squared test was used for statistical comparisons between groups, and a *p* value of <0.05 was taken as significant.

## 3 Results

**Fig. 1:**
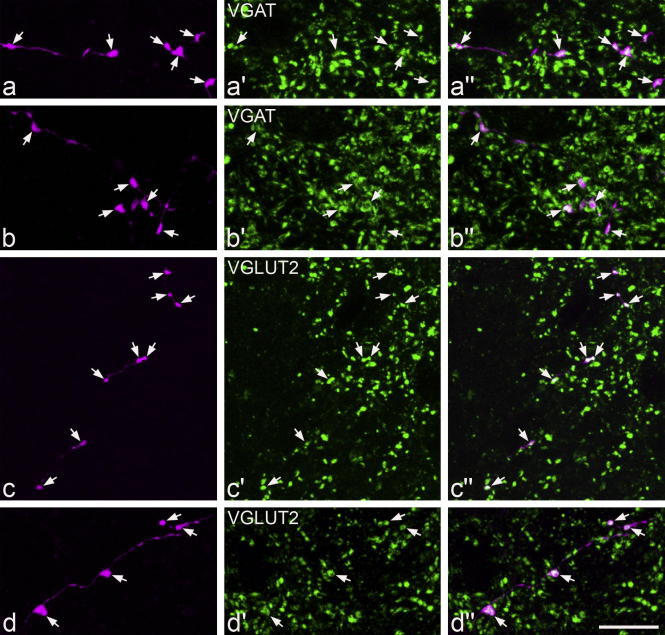
Projected confocal images of immunostaining for VGAT (a and b) or VGLUT2 (c and d) in parts of the axons of 4 lamina II neurons: a an inhibitory vertical cell (i in Fig. 2), b an islet cell (d in Fig. 2), c an unclassified excitatory cell (n in Fig. 3), d an excitatory vertical cell (b in Fig. 3). In each case, the image on the left shows the axon labelled with Neurobiotin and avidin-rhodamine (magenta), with boutons indicated by arrows. The central image shows immunostaining for VGAT or VGLUT2 (green), while a merged image appears on the right. Note that the boutons are labelled with antibody against VGAT (a and b) or VGLUT2 (c and d). The images are projections of 4 (a), 5 (b), 7 (c) or 16 (d) optical sections at 0.3 (a, b and d) or 0.5 (c) μm *z*-spacing. Scale bar = 10 μm.

Altogether 74 neurons that had undergone whole-cell recording were identified histologically by the presence of Neurobiotin labelling. For 67 of these cells the neurotransmitter type was identified as glutamatergic or GABA/glycinergic, by the presence of VGLUT2 or VGAT in axonal boutons. Of these cells, 33 had boutons that were VGAT-immunoreactive and these were defined as inhibitory neurons, while the remaining 34 had VGLUT2-immunoreactive boutons and were defined as excitatory neurons. Examples of each type of immunostaining are shown in Fig. 1. The cell bodies of 61 of the 67 cells for which the transmitter type was identified were clearly located within lamina II. The cell bodies of two neurons (one of which was inhibitory and one excitatory) were 15–18 μm below the dorsal white matter, and were identified as being at the border between laminae I and II. The cell bodies of the other 4 neurons (all of which were VGAT-positive) were located in lamina III.

For the 7 cells for which the transmitter was not identified, no axon was seen in 3 cases, while in the other 4 we were not able to identify either VGLUT2 or VGAT in axonal boutons belonging to the cell. These cells were not analysed further, since the main purpose of this study was to compare morphological, physiological and pharmacological properties of excitatory and inhibitory interneurons.

### 3.1 Morphology of the lamina II neurons

**Fig. 2:**
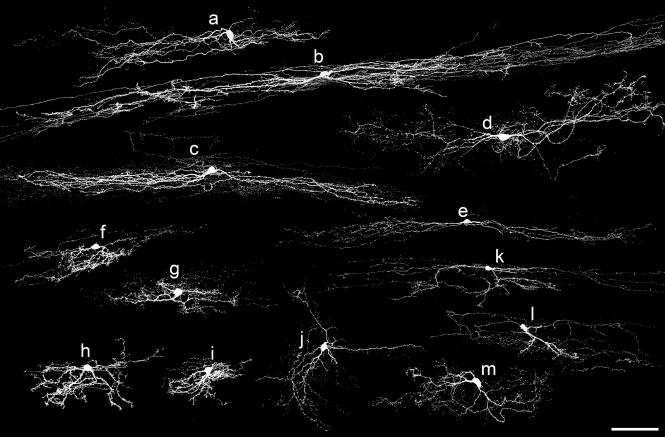
Confocal images showing the cell bodies and dendrites of 13 of the inhibitory neurons. These were classified as: islet cells (a–e), central cells (f and g), and vertical cells (h and i). Cells j–m are unclassified. Note that most of the axon of each cell has been omitted. Scale bar = 100 μm.

**Fig. 3:**
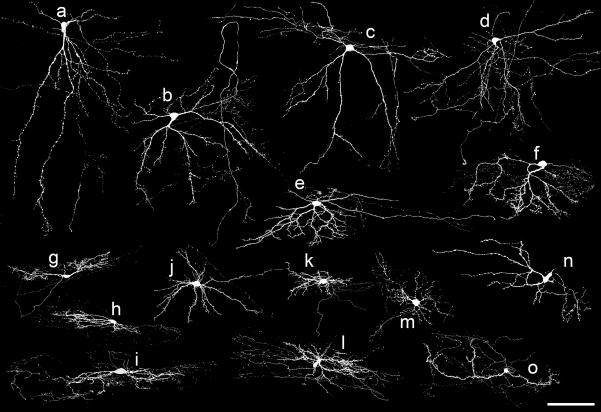
Confocal images showing cell bodies and dendrites of 15 of the excitatory neurons. These were classified as: vertical cells (a–f), central cells (g–i) and radial cells (j–m). Cells n and o are unclassified. Note that most of the axon of each cell has been omitted. Scale bar = 100 μm.

The cells were assessed according to the morphological classification scheme developed by Grudt and Perl [14] and used in most subsequent studies. Examples of cells belonging to different morphological classes are illustrated in Figs. 2 and 3. Of the 61 cells that definitely had their somata in lamina II, 42 were assigned to islet, central, radial or vertical classes (see below). The remaining 19 cells could not be classified, because their morphology was atypical or intermediate between two classes (Fig. 2j–m, 3n and o). Table 1 provides information on the dendritic trees, axonal arbors and soma locations of the cells.

Islet cells (*n* = 12; Fig. 2a–e) were identified by their rostrocaudally elongated dendritic trees, which had a relatively limited dorsoventral spread. Their axons generally arborised within the volume occupied by the dendritic trees and were mainly located in lamina II. However, in some cases they extended further rostrally or caudally than the dendrites, and for some cells they entered lamina I and/or lamina III. The cell bodies of most islet cells were located near the border between laminae IIo and IIi.

Central cells (*n* = 8; Figs. 2f and g, 3g–i) also had dendritic trees that were relatively longer in the rostrocaudal than the dorsoventral axis, but these were considerably smaller than those of the islet cells. Central cell axons arborised in lamina II, but extended beyond the dendritic trees, with branches entering laminae I or III in some cases. Their cell bodies were located throughout the depth of lamina II.

Radial cells (*n* = 7) had dendrites that extended in all directions when viewed in the sagittal plane (Fig. 3j–m). Their dendritic trees were compact, with limited extension in both rostrocaudal (<275 μm) and dorsoventral (<160 μm) axes, and they resembled the radial cells described by Grudt and Perl [14] and by Yasaka et al. [67]. Their axons often travelled for over 500 μm either rostral or caudal to the cell body within lamina II, and in some cases there were branches that entered laminae I and/or III. As with the central cells, somata of radial cells were found throughout the depth of lamina II. One of the other cells (Fig. 2j) also had radiating dendrites, but these were far more extensive (345 and 287 μm in rostrocaudal and dorsoventral axes, respectively), and this cell was therefore assigned to the unclassified group.

Vertical cells (*n* = 15; Figs. 2h and i, 3a–f) differed from radial cells in that their ventrally directed dendrites were particularly prominent, and the cell body generally lay dorsally within the volume occupied by the dendritic tree. Some of these cells had numerous dendritic spines and resembled the stalked cells identified by Gobel [9,10], but other cells with similar morphology had few dendritic spines. Axons of vertical cells arborised in lamina II, with extension into laminae I and/or III in some cases. In most cases, the soma was located in lamina IIo, although a few were in IIi or near the IIo/IIi border.

### 3.2 Morphology and transmitter type

Comparison of the morphology of inhibitory (Fig. 2) and excitatory (Fig. 3) cells revealed certain consistent patterns (Table 1). All of the islet cells were inhibitory (Fig. 2a–e), while all of the radial cells were excitatory (Fig. 3j–m).

**Fig. 4:**
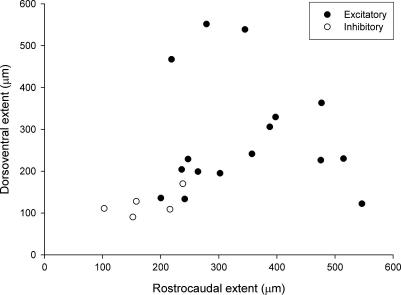
Scatter plot of rostrocaudal and dorsoventral exent of the dendritic trees of excitatory (filled circles) and inhibitory (open circles) vertical cells. Ten of the cells are from the sample reported in this article and the other six are those illustrated in Fig. 1 of Maxwell et al. [32]. Note that excitatory cells generally have larger dendritic trees.

The vertical cells included both excitatory (*n* = 12; Fig. 3a–f) and inhibitory (*n* = 3; Fig. 2h and i) neurons. However, within this group, dendritic trees of the 3 inhibitory cells were among the smallest in both rostrocaudal and dorsoventral dimensions. Maxwell et al. [32] described 6 vertical cells in slices from rat spinal cord, of which 4 were identified as glutamatergic and 2 as GABAergic, and these are illustrated in their Fig. 1. In order to compare a larger sample of excitatory and inhibitory vertical cells, we plotted measurements of the rostrocaudal and dorsoventral dendritic extent of the 15 vertical cells described in the present study and of the 6 vertical cells from Maxwell et al. [32]. This revealed that the dendritic trees of the excitatory cells were generally larger than those of the inhibitory ones (Fig. 4). Axonal projections of excitatory and inhibitory vertical cells in the present sample also differed, in that those belonging to 10 of the 12 excitatory cells entered lamina III, while those of the 3 inhibitory cells did not. The cell bodies of the 3 inhibitory vertical cells were all in lamina IIo, while some of those belonging to the excitatory cells were found in deeper parts of lamina II.

Four of the central cells were inhibitory (Fig. 2f and g) and four were excitatory (Fig. 3g–i). Although the dorsoventral dendritic dimensions of the excitatory cells were somewhat more restricted than those of the inhibitory cells, the sample size was not large enough to allow us to determine whether this represented a genuine difference or whether there were differences in their axonal arborisation patterns or soma locations.

The unclassified group included 9 inhibitory (Fig. 2j–m) and 10 excitatory (Fig. 3n and o) neurons. Dendritic tree dimensions of the two groups overlapped, and both groups contained some cells with axons that entered lamina I and/or lamina III.

### 3.3 Transmitter content and electrophysiology

Electrophysiological data were obtained from 50 lamina II neurons, 23 of which were inhibitory and 27 excitatory. The mean resting membrane potential for the inhibitory neurons (−60.7 ± 1.6 mV SEM) did not differ significantly from that of the excitatory cells (−64.1 ± 1.7 mV) (*t*-test, *p* > 0.05).

**Fig. 5:**
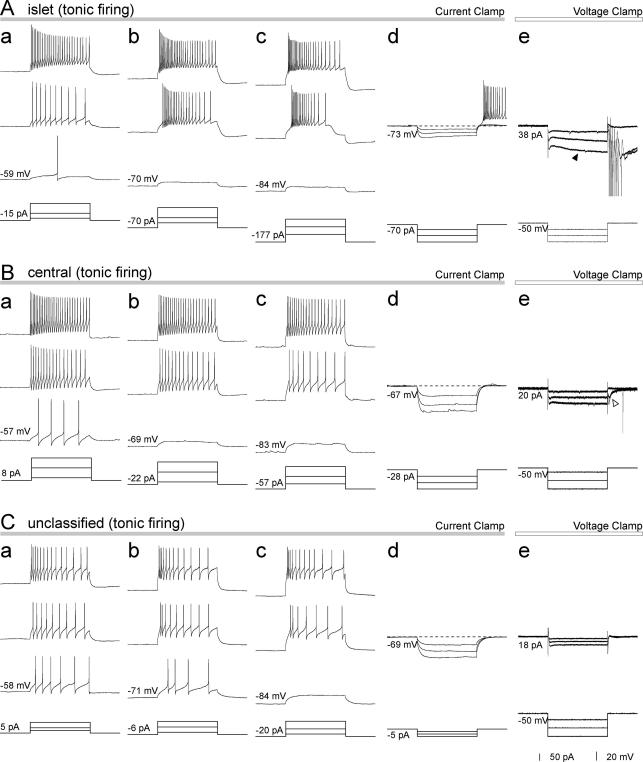
Discharge patterns observed in inhibitory neurons. Examples of the tonic discharge pattern from an islet (A), a central (B) and an unclassified cell (C). Values at the left side of each trace indicate initial membrane voltage or current before application of current or voltage pulses to hold the membrane potential at a certain level. For each cell, in current-clamp mode prior to application of pulses, the membrane potentials were adjusted to within three ranges; a between −50 and −65 mV (in most cases −57 mV); b between −65 and −80 mV (in most cases −72 mV); c more negative than −80 mV (in most cases −87 mV) by continuous current injection. This was done in order to test the voltage dependence of discharge pattern generation. (d) For the test of voltage responses to hyperpolarising current injection, membrane potentials were initially adjusted to −70 ± 3 mV. (e) Ionic currents were tested by applying hyperpolarising voltage steps in voltage-clamp mode from an initial holding potential of −50 mV. The filled arrowhead in Ae indicates hyperpolarising-induced current (*I*_h_), while the open arrowhead in Be indicates transient inward currents observed at the end of hyperpolarising voltage pulses which are probably mediated by low threshold calcium channels (*I*_Ca_). All current or voltage pulses are of 1 s duration.

**Fig. 6:**
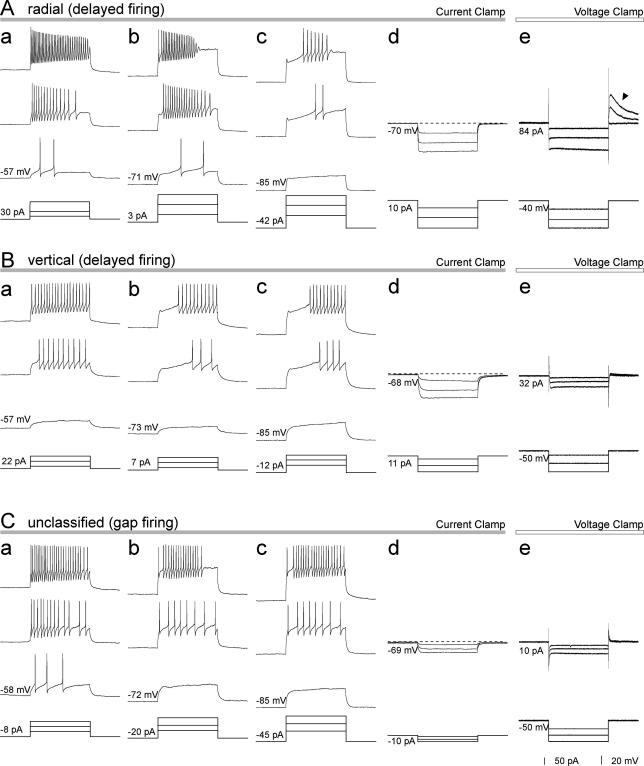
Discharge patterns observed in excitatory neurons. Examples of the delayed discharge pattern from a radial (A) and a vertical (B) cell and of the gap discharge pattern from an unclassified cell (C). Values at the left side of each trace indicate initial membrane voltage or current before application of current or voltage pulses to hold the membrane potential at a certain level. In each case, in current-clamp mode prior to application of pulses, the membrane potentials were adjusted to within three ranges; a between −50 and −65 mV (in most cases −57 mV); b between −65 and −80 mV (in most cases −72 mV); c more negative than −80 mV (in most cases −87 mV) by continuous current injection. This was done in order to test the voltage dependence of discharge pattern generation. (d) For the test of voltage responses to hyperpolarising current injection, membrane potentials were adjusted to −70 ± 3 mV. (e) Ionic currents were tested by applying hyperpolarising voltage steps in voltage-clamp mode from an initial holding potential of −50 mV (or in some cases, −40 mV). The filled arrowhead in Ae indicates transient outward currents with slow kinetics observed at the end of hyperpolarising voltage pulses which are probably mediated by a subclass of A-type potassium channel (*I*_A_) with slow kinetics. All current or voltage pulses are of 1 s duration.

Discharge patterns during 1s periods of current injection were tested in 45 of the neurons. The membrane potential was adjusted to three different levels by continuous current injection prior to the 1s depolarising current pulses. Within our sample we observed discharge patterns including tonic-firing, delayed-firing, gap-firing, transient (initial burst)-firing, single-spiking and reluctant-firing (Figs. 5 and 6) that have been described in previous studies [13,14,17,47,48,68]. A summary of these results is shown in Table 2. Twelve of the cells (2 inhibitory and 10 excitatory neurons) showed differing discharge patterns in response to current injection at different holding potentials. Within this group, 9 showed tonic or transient firing patterns at less negative holding potentials but gap, delayed or reluctant patterns at more negative potentials, while 2 changed from delayed to reluctant or gap to delayed under these conditions. This indicates voltage dependence of a discharge pattern that is probably related to A-type potassium channels, and in these cases the pattern seen at the most negative potential was used to classify the cell [17,47]. One cell changed from transient to single spike at more negative holding potentials, and this was classified as transient.

Delayed-, gap- and reluctant-firing are considered to represent an A-current-related discharge pattern [13,17,47,68]. Cells with these discharge patterns were therefore grouped together for descriptive purposes. In total, 20/45 (44%) of the neurons belonged to this group. However, we found a significant difference in the proportion of inhibitory (2/23, 9%) and excitatory (18/22, 82%) neurons that showed these types of discharge pattern (*p* < 0.005, Chi-squared test). All of the radial cells had an A-current-related pattern, and within the vertical group the presence of this pattern was closely related to neurotransmitter type, being found in all of the glutamatergic, but none of the GABAergic cells. Although we did not carry out a detailed analysis of voltage-activated currents following release from hyperpolarising voltage steps, these were examined in most neurons. Both slow and fast *I*_A_ currents were observed, and these were restricted to excitatory cells with one exception, an inhibitory neuron that showed a mixed current (probably resulting from of the presence of both *I*_A_ and *I*_Ca_). However, the amplitude of the fast *I*_A_ currents was often very small (∼10 pA) and difficult to distinguish. These results, together with voltage responses to hyperpolarising currents, are illustrated in supplemental Figs. 1 and 2. No clear relationship between the type of *I*_A_ current (fast or slow) and firing pattern was observed, except that all of the 6 cells with slow *I*_A_ currents showed a tonic or transient (i.e. a non-*I*_A_ type) firing pattern when held at around -57 mV. In contrast, 11 of the 12 cells with fast *I*_A_ currents, as well as 2 cells that showed delayed-firing but did not have detectable *I*_A_ currents, also had *I*_A_-type firing patterns when held at ∼ -57 mV. This is consistent with the report by Ruscheweyh et al. [47] that removal of steady-state inactivation required more negative holding potentials for the slow *I*_A_ current than for the fast one. Of the 18 excitatory cells that had *I*_A_-type firing patterns, 17 were tested for the presence of *I*_A_ currents. Fifteen of these were found to have *I*_A_ currents (10 fast, 5 slow), while one had a mixed current and one had no detectable current. Three additional excitatory neurons showed *I*_A_ currents (one slow, two fast), but their firing patterns were not tested.

Currents induced by hyperpolarising voltage steps (*I*_h_) were also tested (Supplemental Fig. 3). These were measured at a holding potential of −100 mV to minimise the contribution from inward potassium currents below their reversal potentials. We found pronounced *I*_h_ (inward currents larger than −40 pA) in 5 out of 21 inhibitory neurons but in none of 24 excitatory cells.

### 3.4 Neurotransmitter phenotype and pharmacology

**Fig. 7:**
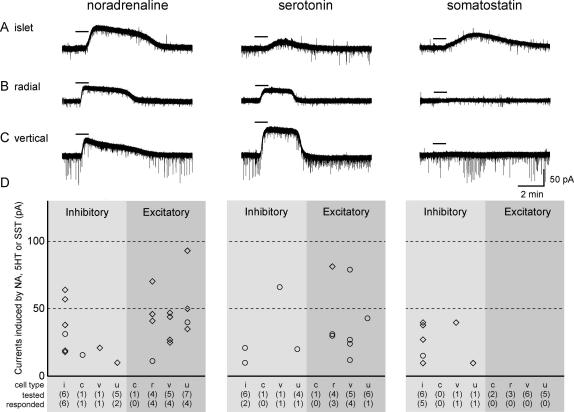
Responses to neuromodulators observed in lamina II neurons. Traces obtained from an islet cell (A), a radial cell (B) and an excitatory vertical cell (C) demonstrate responses during a series of applications of noradrenaline (NA), serotonin (5HT) and somatostatin (SST). Horizontal bars indicate 1 min applications of neuromodulators. (D) The amplitudes of outward currents in response to drug application for each of the inhibitory and excitatory cells tested, grouped according to morphological class. Diamonds show responses to 40 μM NA, 40 μM 5HT or 2 μM SST, while circles show responses to 20 μM NA, 20 μM 5HT or 1 μM SST. For each cell, only one concentration of each neuromodulator was tested. Note that most (7/8) of the inhibitory cells, but none of the excitatory cells, responded to somatostatin. c, central; i, islet; r, radial; u, unclassified; v, vertical.

Responses to application of NA, 5HT and/or somatostatin were tested on a sample of the recorded neurons. All 3 drugs caused an outward current in a proportion of the cells tested (Fig. 7), while inward currents were not seen in response to application of any of these drugs. Spontaneous events were not analysed in this study. NA was tested on 30 cells and caused outward current in 22 of them (73%), while for 5HT and somatostatin the proportions that showed outward currents were 12/28 (43%) and 7/24 (29%), respectively. Positive responses were defined as currents that were larger than 10 pA.

The proportion of excitatory (12/17, 71%) and inhibitory (10/13, 77%) neurons showing outward currents in response to NA was very similar. When the effects of NA on different morphological types were compared, it was found that all islet cells (6/6), all radial cells (4/4) and most excitatory vertical cells (4/5) responded (Fig. 7D). The proportion of inhibitory neurons (4/12, 33%) showing outward currents in response to 5HT was smaller than that of excitatory ones (8/16, 50%), although this difference was not significant (*p* = 0.6, Chi-squared test). One third of the islet cells (2/6), most radial cells (3/4) and most excitatory vertical cells (4/5) responded to 5HT. In the sample tested with NA and 5HT, most radial cells (3/4) and all excitatory vertical cells (4/4) exhibited responses to both drugs while only some islet cells (2/6) did (Fig. 7D).

Consistent with previous reports [21,23], we found that somatostatin induced outward currents in a proportion (7/24, 29%) of the lamina II neurons tested, and here there was a clear difference between neurotransmitter types. Neurons that responded to somatostatin were limited to the inhibitory population (7/8, 88%), while none of the 16 excitatory cells that were tested responded (Fig. 7D). Within the limited sample of cells tested, 5 out of 6 islet cells were found to respond to somatostatin. Seven of the cells that responded to somatostatin were tested with NA and 5HT: 3 of these responded to NA but not 5HT, 1 to 5HT but not NA and 3 to both monoamines.

### 3.5 Somatostatin immunostaining

**Fig. 8:**
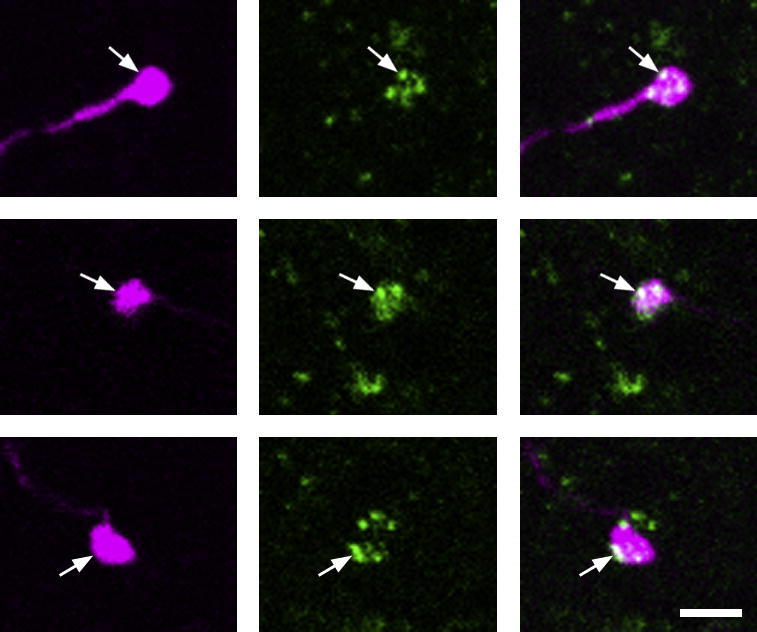
Somatostatin immunoreactivity in axonal boutons belonging to one of the excitatory interneurons. The figure shows 3 boutons from one of the radial cells (cell m in Fig. 3) in a section that had been reacted to reveal Neurobiotin (magenta) and somatostatin (green). In each field, several small patches of somatostatin immunoreactivity are visible, and some of these are contained within the bouton (one is indicated with an arrow in each bouton). Somatostatin immunoreactivity outside the labelled boutons represents expression of the peptide by nearby axons belonging to other cells. Images were obtained from two optical sections 0.3 μm apart (top row) or from a single optical section (middle and bottom rows). Scale bar = 2 μm.

In the sections reacted with somatostatin antibody, immunoreactivity was seen at high levels in the superficial dorsal horn, where it was found in numerous small structures that resembled axonal boutons, as well as in some cell bodies. Fourteen of the twenty-four excitatory cells tested had somatostatin-immunoreactive boutons, and within these boutons the immunostaining appeared in small clumps that probably correspond to clusters of dense-cored vesicles [55] (Fig. 8). Table 3 shows the morphology and firing patterns of cells that were tested for somatostatin immunoreactivity. All five of the radial cells tested were somatostatin positive, as were half of the vertical and central cells, and 3 of 7 unclassified cells. The somatostatin-positive group included cells b, c, d, e, h, k, m and o from Fig. 3, while the somatostatin-negative group included cells a, f and n from this figure. No obvious morphological differences were seen between somatostatin-positive or -negative cells among the vertical or unclassified populations.

Firing patterns were investigated for 15 of the 24 cells that were tested for somatostatin immunoreactivity. Most (7/10) of the cells with delayed-firing, but none of the cells with other firing patterns, were somatostatin positive (Table 3). Although the sample of cells with gap, reluctant, transient and tonic patterns is too small to allow interpretation, this result suggests that many somatostatin-containing cells will have delayed-firing patterns.

## 4 Discussion

The major findings of this study are that: (1) although excitatory and inhibitory interneurons show considerable heterogeneity, certain morphological types are consistently found in each group; (2) most excitatory neurons, but few inhibitory cells, have delayed-, gap- and reluctant-firing patterns; (3) although hyperpolarizing actions of NA and 5HT are seen in both groups, those of somatostatin are restricted to inhibitory neurons; (4) somatostatin is present in various morphological types of glutamatergic interneuron, including cells with delayed-firing.

### 4.1 Classification of lamina II neurons

Whole-cell recording studies have identified four main neuronal classes in lamina II: islet, vertical, radial and central cells [14,17,28–30,32,67,69]. There has been variation in the proportions assigned to each class, for example Grudt and Perl [14] classified 29% of their sample as central cells, while these constituted 13% in our study. Although this variation could be due to species differences, it is more likely to result from technical issues, such as plane of section and whether cells were visually targeted. Both targeted and blind recording techniques have a sampling bias. With blind recording, larger cells are more likely to be selected, which could account for the relatively few central cells in this study and the higher than expected proportion of inhibitory interneurons (46%, rather than 31% [40]). However, visually targeted recording can only be performed on cells located superficially within the slice and favours those with compact dendritic trees. Despite the inevitable bias, our sample contained cells in each of the main classes.

Islet cells [9], have elongated dendritic trees and characteristic physiological properties [14,33,67]. Gobel et al. [11] proposed that they were inhibitory and this was confirmed by the demonstration that they were GABA immunoreactive [58,61]. The 5 islet cells examined by Maxwell et al. [32] had GAD-containing axons, and all 12 in our sample were VGAT positive. Physiological studies have shown that islet cells form GABAergic synapses [28,69], and these cells therefore constitute a recognisable population of inhibitory interneurons.

Gobel [10] classified cells with ventrally directed spine-covered dendrites and axons entering lamina I as stalked cells, and proposed that they were excitatory. Consistent with this, we reported that stalked cells were not GABA immunoreactive [58]. However, Grudt and Perl [14] observed similar cells with few dendritic spines and axons that did not enter lamina I, and included them in a larger population of vertical cells. Our results demonstrate that while most vertical cells are excitatory, there is a distinctive population of inhibitory neurons that are morphologically similar, although having smaller dendritic trees and different firing patterns. This indicates that caution is needed when interpreting the role of vertical cells in neuronal circuits [69].

We observed a population of radial cells with compact dendritic trees, similar to those described in previous studies [14,67]. These were glutamatergic and showed *I*_A_-type firing patterns. However, they must be distinguished from cells with much longer radiating dendrites, some of which are GABAergic [32,58].

Todd and McKenzie [58] identified a group of non-GABA-immunoreactive neurons resembling islet cells, but with shorter dendritic trees. Grudt and Perl [14] classified neurons of this type as central cells, which they divided into tonic, transient *I*_A_ and transient non-*I*_A_ classes. Transient non-*I*_A_ and tonic central cells can form glutamatergic synapses onto vertical cells [28,29]. However, Perl’s group [16,69] have identified a population of GABAergic tonic central cells that are presynaptic to vertical and islet cells, while Maxwell et al. [32] observed an inhibitory central cell. Consistent with these reports, we identified both GABAergic and glutamatergic central cells.

It is possible that for central cells and for those currently unclassified, there are further classes still to be identified. Alternatively, these may represent morphologically heterogeneous populations of excitatory and inhibitory interneurons. The first interpretation is suggested by the finding of small but distinctive populations, such as inhibitory central cells that express green fluorescent protein (GFP) under control of the prion promoter [16,69]. Several neuropeptides and proteins have been identified among superficial dorsal horn neurons [59]. Although some of these, such as somatostatin, are expressed by numerous cells, others show a more restricted distribution. For example, NPY, galanin, parvalbumin and nitric oxide synthase (NOS) are found in non-overlapping populations of GABAergic neurons in laminae I–III [25] (Todd et al., unpublished observations). Some of these populations innervate particular classes of projection neuron: neurokinin 1 receptor-expressing lamina III projection cells receive numerous synapses from NPY/GABA axons [41], while another type of projection neuron in lamina I is densely innervated by NOS/GABA axons [39,44]. Combining neurochemical identification with the approach used in this study should help to refine our classification of lamina II interneurons [12].

### 4.2 Firing patterns associated with *I*_A_ currents

*I*_A_ currents control neuronal excitability by delaying the first action potential and reducing discharge frequency, giving rise to delayed-, gap- or reluctant-firing. In superficial dorsal horn they are mediated by Kv4-containing channels, with Kv4.2 playing a particularly important role [18]. Previous studies have identified these patterns and shown their association with particular cell types [13,14,42,47]. However, ours is the first to demonstrate directly that *I*_A_-type firing patterns are largely restricted to excitatory interneurons and associated with most of these cells. This extends the findings of Heinke et al. [17], who investigated mice in which GFP is expressed by some GABAergic lamina II neurons and found that delayed and gap patterns were mainly associated with GFP-negative cells. They are also consistent with expression of Kv4.2 and Kv4.3 by calretinin-containing and μ-opioid receptor-immunoreactive lamina II neurons [19], as these are thought to be glutamatergic [2,22].

Loss of *I*_A_ currents in *Kv4.2*^−/−^ mice causes increased excitability and firing frequency, resulting in heightened sensitivity to tactile and thermal stimuli [18]. Although Ruscheweyh et al. [47] reported gap-firing lamina I spinoparabrachial neurons, it is unlikely that these contribute to this behavioural phenotype, since Kv4.2 is expressed at very low levels in lamina I [19]. Our results strongly suggest that excitatory lamina II interneurons with *I*_A_-type firing patterns have an important role in synaptic plasticity in inflammatory pain states, since Kv4.2 is a down-stream target for phosphorylation by extracellular signal-regulated kinases (ERKs) [18], which are activated in many lamina II neurons by noxious stimuli [20].

Together with the findings of Heinke et al. and Ruscheweyh et al. [17,47], our results demonstrate the importance of testing firing patterns from different holding potentials. The use of only a single holding potential in previous studies may account for some of the differences in firing patterns reported by different groups.

### 4.3 Responses to neuromodulators

Our results with NA and 5HT, which are consistent with those of previous studies [1,15,30,38], suggest that there is unlikely to be a major difference between the proportions of inhibitory and excitatory interneurons that are hyperpolarised by the two monoamines.

In contrast, there was a clear difference in responses to somatostatin, which were only seen with inhibitory interneurons. Somatostatin is synthesised by primary afferents and many glutamatergic interneurons in laminae I–II [43,55], released upon noxious stimulation [24], and acts on sst2a receptors [51,52] to hyperpolarise some neurons in this region [21,23]. Our findings are compatible with immunocytochemical data showing that sst2a is restricted to GABA-immunoreactive neurons in lamina II [60]. Although high doses of somatostatin are anti-nociceptive these may be neurotoxic [8,36], and at physiological levels intrathecal somatostatin appears to be pro-nociceptive [53,63,64], consistent with a disinhibitory effect involving hyperpolarisation of inhibitory interneurons.

**Fig. 9:**
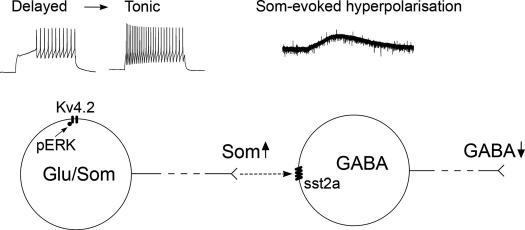
Proposed mechanism for involvement of somatostatin-evoked disinhibition in Kv4.2-mediated synaptic plasticity in the superficial dorsal horn. Noxious stimulation leads to production of phosphorylated ERK (pERK) in somatostatin-containing excitatory lamina II interneurons, which in turn phosphorylates the Kv4.2 channel (Hu et al. [18]). This results in an increase in excitability of these neurons, which may be associated with a change from a delayed- to a tonic-firing pattern, and a consequent increase in release of somatostatin in response to peripheral stimulation. The somatostatin acts through volume transmission on sst2a receptors expressed by nearby inhibitory interneurons, causing hyperpolarisation and a decrease of their excitability, which leads to a reduction of GABA release (disinhibition).

Nakatsuka et al. [37] demonstrated a slow outward current in ∼30% of lamina II neurons evoked by focal stimulation, and substantially reduced by a somatostatin receptor antagonist. Dorsal root stimulation failed to produce this current, presumably because most somatostatin-containing axons in this region originate from glutamatergic interneurons [43,49,55]. Together with the present findings, this suggests that somatostatin released from these cells can hyperpolarise a subpopulation of GABAergic neurons in lamina II. Most somatostatin-containing interneurons showed delayed-firing, indicating the presence of *I*_A_ currents, and we have found Kv4.2 immunoreactivity associated with somatostatin-immunoreactive lamina II neurons (Todd, unpublished observations). Noxious stimulation may activate ERKs in these cells [20], leading to Kv4.2 phosphorylation, and inhibition of *I*_A_ currents. The resulting increase in their excitabilty could lead to enhanced activation following peripheral stimulation, and thus increase somatostatin release, causing hyperpolarisation of nearby inhibitory interneurons (Fig. 9). A disinhibitory mechanism involving somatostatin may therefore contribute to Kv4.2-mediated plasticity in the superficial dorsal horn [18].

### 4.4 Conclusions

Although the neuronal organisation of lamina II is complex, our results together with those of previous studies [14,16,17,28,29,32,67], show that certain distinctive populations of lamina II interneurons can be recognised, for example islet cells, which are inhibitory, and radial and large vertical cells, which are excitatory. However, it is important to note that some neurons with the morphological appearance of vertical cells are GABAergic. Our finding that firing patterns associated with *I*_A_ currents are largely restricted to excitatory interneurons, suggests that these have an important role in ERK-mediated central sensitisation and pain plasticity involving Kv4.2 [18]. Finally, we have identified a potential disinhibitory mechanism involving somatostatin, which is released by several types of excitatory interneuron and hyperpolarises inhibitory interneurons.

## 5 Conflicts of interest statement

The authors report no conflicts of interest.

## Figures and Tables

**Table 1:** Morphological features of different types of inhibitory and excitatory lamina II neuron.

Transmitter type	Morphological type	Number	Dendritic dimensions (μm)		Axonal arbor			Soma location		
			Rostrocaudal	Dorsoventral	I	II	III	IIo	IIo/i	IIi
Inhibitory (*n* = 28)	Islet	12	466–1069 (731)	42–176 (108)	6	12	5	1	10	1
	Central	4	191–385 (294)	71–89 (76)	1	4	1	1	2	1
	Vertical	3	103–216 (157)	90–111 (103)	1	3	0	3	0	0
	Unclassified	9	144–1639 (520)	68–246 (135)	4	9	3	3	2	4
Excitatory (*n* = 33)	Central	4	189–324 (249)	49–70 (57)	3	4	2	1	1	2
	Radial	7	147–271 (203)	59–157 (118)	4	7	4	2	3	2
	Vertical	12	200–546 (370)	122–552 (300)	9	12	10	7	3	2
	Unclassified	10	87–407 (221)	21–299 (130)	8	10	7	2	3	5

The table shows the range of dendritic tree dimensions for each type (mean in brackets), together with the number of cells that had at least some of their axonal arbor in laminae I, II and/or III, and the number with their soma in each part of lamina II. Note that all cells had axons that arborised in lamina II.

**Table 2:** Firing pattern of different types of lamina II neuron.

Inhib/Excit	Morphology (No. tested)	No. showing pattern	Firing pattern		
			(from initial holding potential)		
			−50 to −65 mV	−65 to −80 mV	−80 to −95 mV
Inhibitory	Islet (11)	8	Tonic	Tonic	Tonic
		2	Transient	Transient	Transient
		1	Tonic	Tonic	Gap
	Central (3)	3	Tonic	Tonic	Tonic
	Vertical (2)	2	Tonic	Tonic	Tonic
	Unclassified (7)	6	Tonic	Tonic	Tonic
		1	Transient	Transient/gap	Transient/delayed
Excitatory	Central (1)	1	Tonic	Tonic	Tonic
	Radial (6)	3	Delayed	Delayed	Delayed
		1	Gap	Delayed	Delayed
		1	Tonic	Delayed	Delayed
		1	Tonic	Transient/gap	Transient/delayed
	Vertical (6)	4	Delayed	Delayed	Delayed
		1	Delayed	Delayed	Reluctant
		1	Reluctant	Reluctant	Reluctant
	Unclassified (9)	2	Tonic	Tonic	Tonic
		1	Tonic	Tonic	Gap
		2	Tonic	Gap	Gap
		1	Transient	Transient	Reluctant
		1	Transient	Transient	Single
		1	Transient	Transient/gap	Delayed
		1	Delayed	Delayed	Delayed

**Table 3:** Morphology and firing pattern of cells that were tested for somatostatin-immunoreactivity.

	Morphology				Firing pattern					
	Central	Radial	Vertical	Unclassified	Delayed	Gap	Reluctant	Transient	Tonic	Not tested
Somatostatin-positive	1	5	5	3	7	0	0	0	0	7
Somatostatin-negative	1	0	5	4	3	2	1	1	1	2
Total	2	5	10	7	10	2	1	1	1	9
