# Supplementary material for: Populations of inhibitory and excitatory interneurons in lamina II of the adult rat spinal dorsal horn revealed by a combined electrophysiological and anatomical approach
Source: Pain. 2010 Nov;151(2):475–88. doi: 10.1016/j.pain.2010.08.008 (PMC3170912; doi:10.1016/j.pain.2010.08.008)
Supplement: Supplementary data 1 [file mmc1.doc]

**SUPPLEMENTARY ON-LINE MATERIAL**

**Supplemental Figure 1**


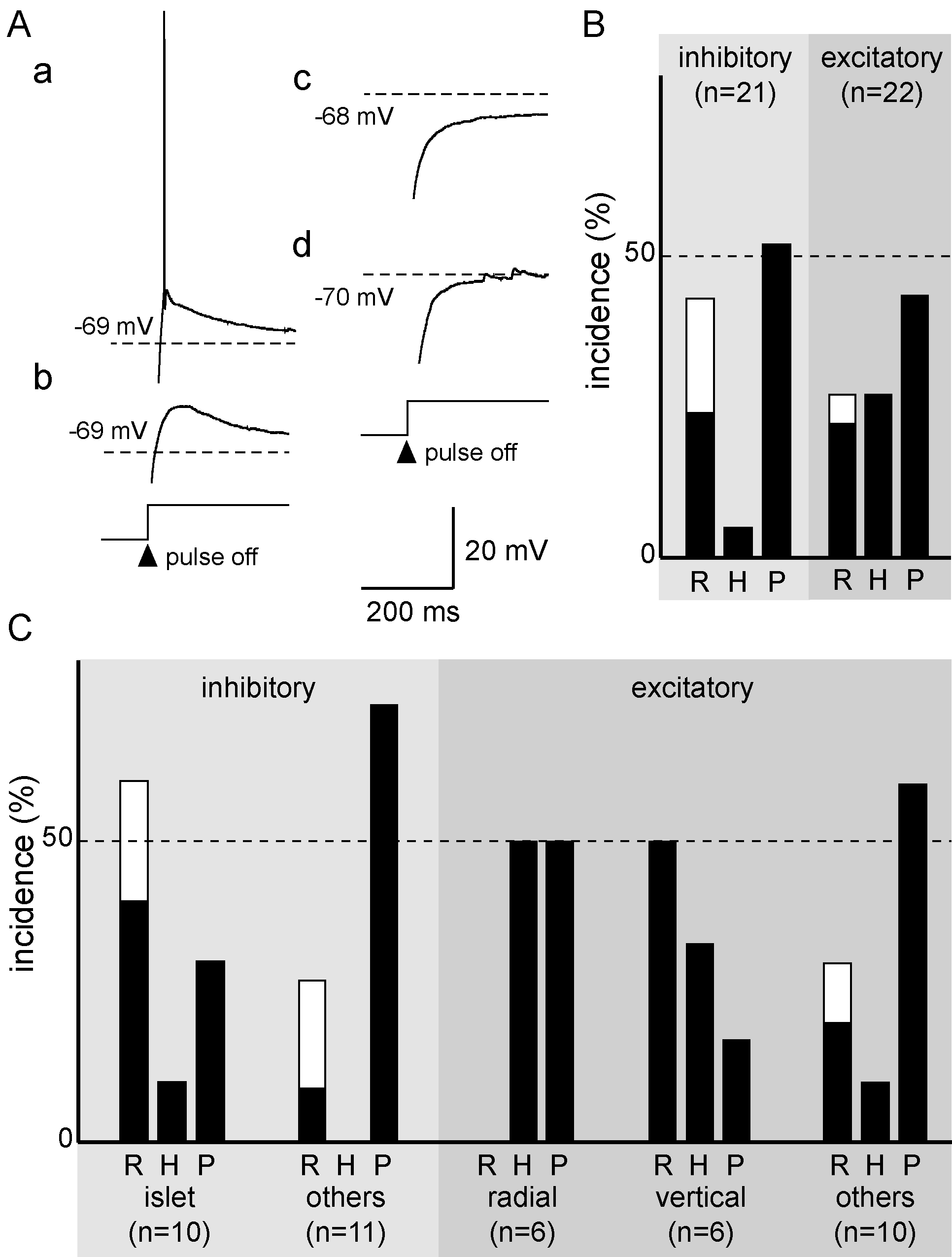


Responses to hyperpolarizing current injection observed in lamina II neurons. **Aa**-**Ad**: traces showing different voltage responses following release from hyperpolarization in current-clamp mode. Responses were classified in accordance with the results of Graham et al. (2007) J Physiol 584:121-136. **Aa**, rebound depolarization with action potential discharge; **Ab**, rebound depolarization without action potential discharge; **Ac**, long-lasting hyperpolarizing response; **Ad**, passive response. Values at the left side of each trace indicate initial membrane voltage before application of current pulses to hold the membrane potential at a certain level (-70 ± 3 mV). The time scale of these traces is expanded compared with those shown in column **d** of Figs. 5 and 6, in order to focus on the time point of release from hyperpolarization, which is shown at the bottom of **b** and **d**. **B**: Incidence of different types of response to hyperpolarization among inhibitory and excitatory cells. Note that for the inhibitory neurons (17 out of 21 of which had a tonic firing pattern) the proportion showing each type of response is very similar to that reported for tonic firing neurons by Graham et al. (2004) J Physiol 561:749-763. **C:** Incidence of response profiles to hyperpolarization grouped according to morphological class. The group "others" in inhibitory cells includes 3 central, 2 vertical and 5 unclassified cells. The "others" group of excitatory cells includes 1 central and 9 unclassified cells. The incidence of responses varies among the excitatory cells. For example, none of radial cells, but half of the vertical cells, show rebound depolarization. R, rebound depolarization with (open bar) and without (filled bar) discharge of action potentials; H, long-lasting hyperpolarizing response; P, passive response.

**Supplemental Figure 2**


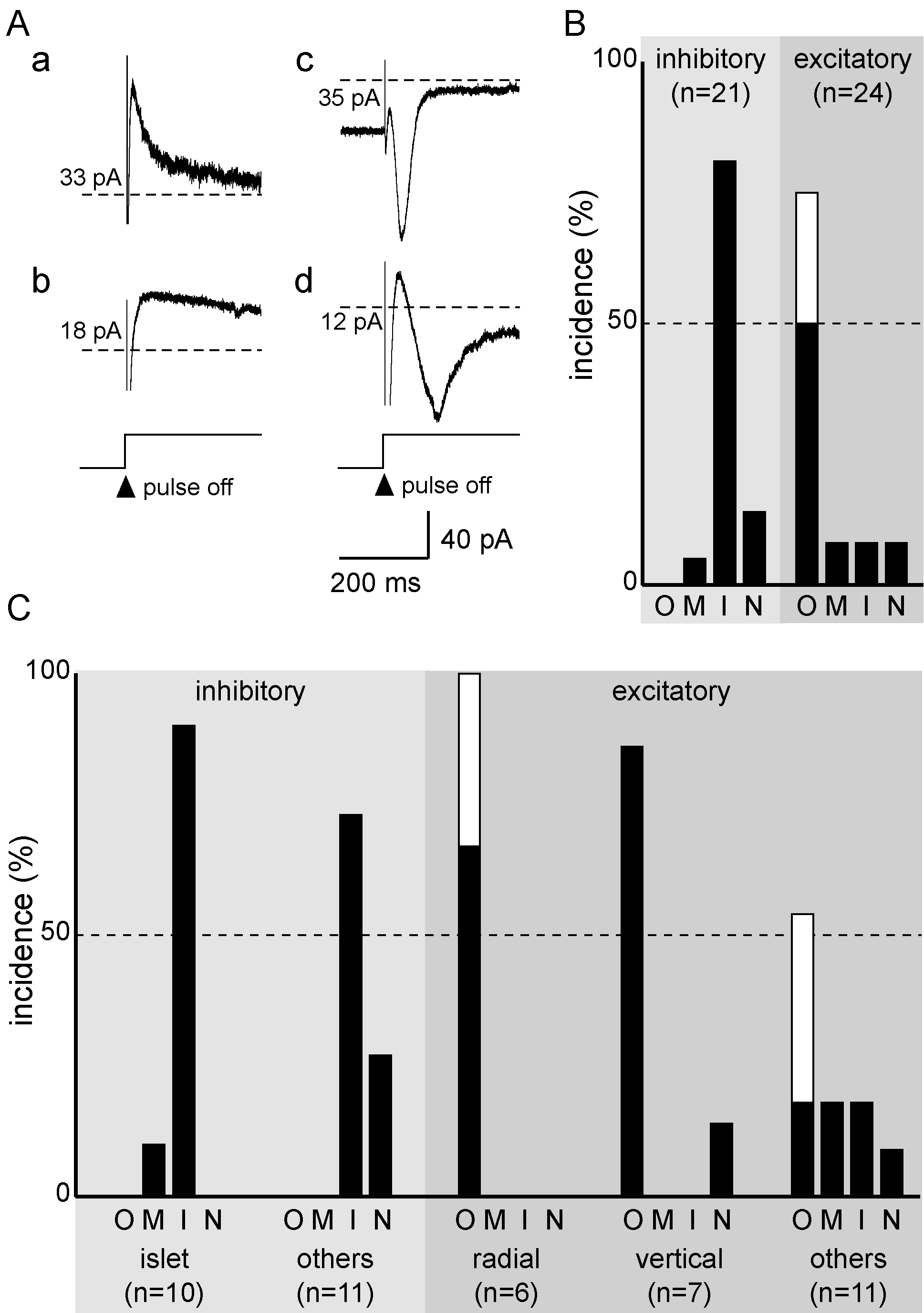


Voltage-activated currents observed in lamina II neurons after release from hyperpolarizing voltage steps. **Aa**-**Ad**: traces showing different responses following release from hyperpolarization in voltage-clamp mode. Response profiles were classified in accordance with those shown by Graham et al. (2007) and Ruscheweyh et al. (2004) J Physiol 555:527-543. **Aa**, a transient fast outward current observed at the end of the hyperpolarizing voltage pulse that is probably mediated by a subclass of the A-type potassium channel (*I*_A_ channel) with fast kinetics; **Ab**, a transient slow outward current induced by the same protocol that is probably mediated by another subclass of *I*_A_ channel with slow kinetics; **Ac**, a transient inward current that is probably mediated by a low threshold calcium channel (*I*_Ca_); **Ad**, a mixed response with outward and inward currents. Values at the left side of each trace indicate initial membrane current to maintain holding potential at -50 mV before application of voltage pulses. The time scale of these traces is expanded compared with those in column **e** of Figs. 5 and 6, in order to focus on the time point of release from hyperpolarization which is shown at the bottom of **b** and **d**. **B**: Incidence of different types of response to hyperpolarization in inhibitory and excitatory cells. Note, there is an obvious difference between inhibitory and excitatory cells, since most inhibitory cells show inward currents while most excitatory cells have outward currents. **C**: Incidence of response profiles to hyperpolarization grouped according to morphological classes. The group "others" in inhibitory cells includes 3 central, 2 vertical and 5 unclassified cells. The "others" group of excitatory cells includes 2 central and 9 unclassified cells. The amplitudes of fast *I*_A_ currents were often very small, such that they were difficult to distinguish. This is due to incomplete activation of *I*_A_ channels at the holding potentials (-50 or -40 mV) that were used in this study (Ruscheweyh et al., 2004). In 2 of the 3 cases of mixed currents, inward currents came first and were followed by outward currents with slow kinetics, while in the third case (Ad) the order was reversed. O, a transient outward current with fast (filled bar) and slow (open bar) kinetics. These currents are probably mediated by different subclasses of *I*_A_ channels; M, mixed responses which contain both outward and inward currents; I, a transient inward current which is probably mediated by low threshold calcium channel; N, no detectable currents.

**Supplemental Figure 3**


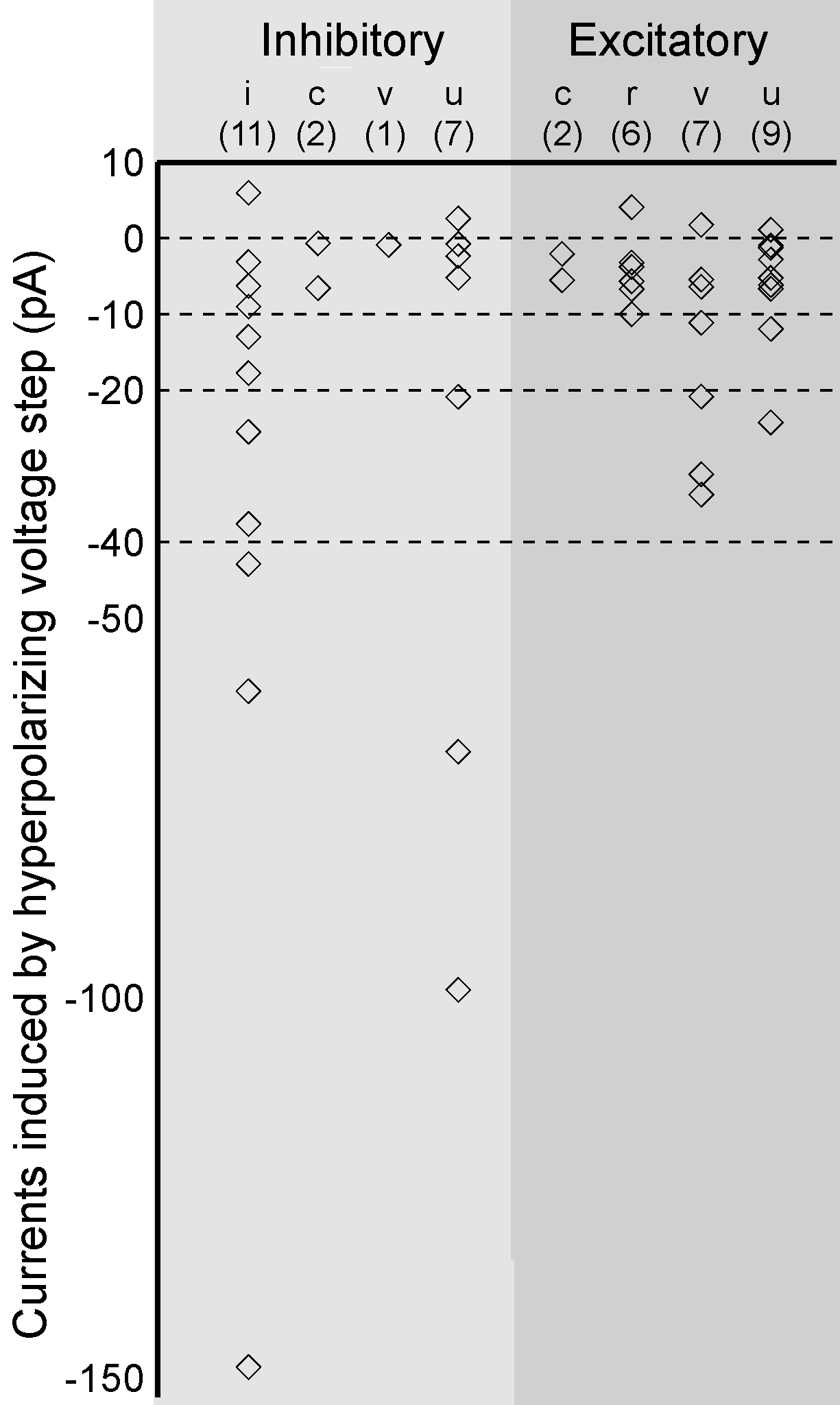


The incidence of *I*_h_ currents in inhibitory and excitatory neurons. The amplitude of *I*_h_ was measured during 1 s hyperpolarizing voltage step from -50 to -100 mV. Values measured immediately after application of the hyperpolarizing pulse were subtracted from those at the end of the pulse to calculate *I*_h_. Currents < 10 Pa are considered to be insignificant. Currents range from small (10-20 pA), through medium (20-40 pA) to large (>40 pA). Note that while *I*_h_ currents are seen in both types of cell, the large currents are restricted to inhibitory neurons. c, central; i, islet; r, radial; u, unclassified; v, vertical.
